# Massive Ovarian Edema Successfully Treated Through Accurate Preoperative Diagnosis and Laparoscopic Conservative Surgery

**DOI:** 10.7759/cureus.98762

**Published:** 2025-12-08

**Authors:** Marina Yagi, Takeshi Fukuda, Takuma Wada, Makoto Yamauchi, Toshiyuki Sumi

**Affiliations:** 1 Department of Obstetrics and Gynecology, Osaka Metropolitan University Graduate School of Medicine, Osaka, JPN

**Keywords:** conservative surgery, fertility preservation, laparoscopy, massive ovarian edema, ovarian torsion

## Abstract

Massive ovarian edema (MOE) is a rare benign condition that causes marked ovarian enlargement due to stromal fluid accumulation and can mimic an ovarian neoplasm. Accurate preoperative diagnosis is essential to avoid unnecessary oophorectomy, especially in young women.

A 26-year-old nulligravid woman presented with right lower abdominal pain. Ultrasonography showed a markedly enlarged right ovary with peripheral follicles and mottled hypoechoic areas. MRI demonstrated an 85-mm enlarged ovary with high T2 signal intensity and multiple peripheral cysts, findings suggestive of MOE. Tumor markers were within normal limits. Based on characteristic imaging features and prior clinical experience, MOE was strongly suspected preoperatively. Laparoscopy revealed a markedly enlarged ovary with a 360-degree torsion. Following detorsion, the ovary decreased in size. A cortical incision revealed no tumorous lesion, and serous fluid leaked from the stroma. A wedge biopsy was performed, and histopathology confirmed MOE. The postoperative course was uneventful, and follow-up ultrasonography one month later showed complete return of the ovary to normal size.

This case illustrates the diagnostic value of characteristic ultrasonographic and MRI findings of MOE and highlights the importance of clinical experience in avoiding unnecessary oophorectomy and achieving fertility-preserving management.

## Introduction

Massive ovarian edema (MOE) is a rare, tumor-like gynecological condition characterized by marked enlargement of the ovary due to the accumulation of edematous fluid within the ovarian stroma, first described by Kalstone et al. in 1969 [1]. This condition predominantly affects adolescents and young women, with a mean reported age of approximately 20 years; however, both premenarcheal [2] and postmenopausal women [3] may also be affected. MOE is generally considered to result from partial or intermittent torsion of the ovarian pedicle, leading to impaired venous and lymphatic drainage while preserving arterial inflow [1]. 

Clinically and radiologically, MOE often mimics a solid ovarian neoplasm, creating a diagnostic challenge. Ultrasonography and computed tomography may show nonspecific ovarian enlargement, whereas magnetic resonance imaging has been reported to reveal characteristic findings such as peripheral displacement of follicles around a centrally edematous stroma-features that may assist in distinguishing this entity from true ovarian tumors [4,5]. Because MOE is a benign and potentially reversible condition, misdiagnosis may lead to unnecessary oophorectomy, resulting in avoidable loss of ovarian function. Fertility-preserving, conservative surgical management is therefore recommended whenever malignancy can be reasonably excluded.

Accurate preoperative recognition is essential, particularly in young women presenting with solid-appearing ovarian enlargement. The present report describes a case of MOE in a 26-year-old woman that was successfully managed with conservative laparoscopic surgery following appropriate preoperative diagnosis.

## Case presentation

**Figure 1:**
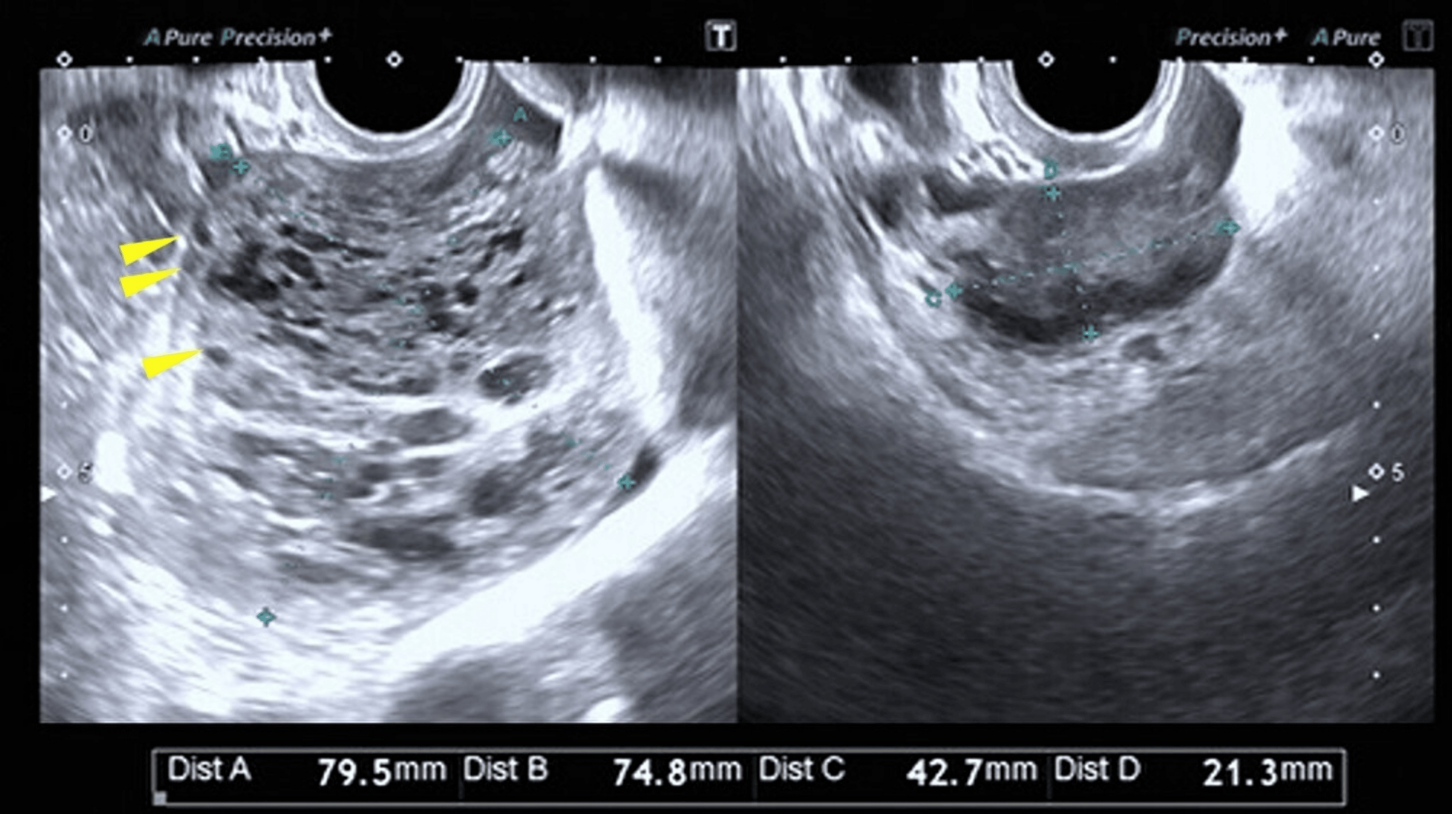
Transvaginal ultrasonography of both ovaries. The image on the left shows the right ovary, measuring 79.5 × 74.8 mm, with multiple small cystic structures along the inner margin and mottled hypoechoic areas within the parenchyma. Arrowheads indicate the small cystic structures. The image on the right shows the normal left ovary, measuring 42.7 × 21.3 mm.

A 26-year-old nulligravid woman with no significant past medical or family history and without any gynecological symptoms other than right lower abdominal pain presented to her previous physician with right lower abdominal pain. Transvaginal ultrasonography performed at the prior clinic revealed a right ovarian mass, and she was subsequently referred to our hospital for further evaluation. At the initial visit, transvaginal ultrasonography demonstrated an enlarged right ovary measuring 79.5 × 74.8 mm, with multiple small cystic structures aligned along the inner margin and mottled hypoechoic areas within the parenchyma (Figure 1).

**Figure 2:**
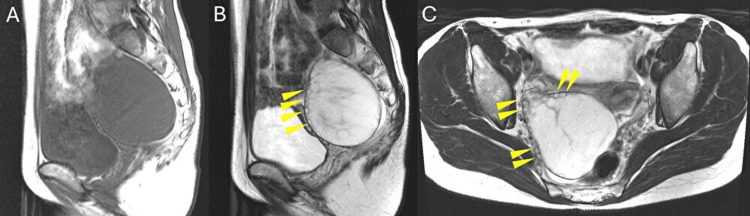
Pelvic MRI findings. (A) T1-weighted sagittal image. (B) T2-weighted sagittal image. (C) T2-weighted axial image. The enlarged right ovary measures 77.9 × 68.7 × 81.0 mm and contains multiple peripheral ovarian follicles. The lesion is isointense on T1-weighted imaging and hyperintense on T2-weighted imaging, consistent with edematous stroma. Arrowheads indicate the small cystic structures.

Pelvic magnetic resonance imaging (MRI) further revealed a 77.9 × 68.7 × 81.08 mm enlarged right ovary, again showing numerous small peripheral cystic structures without any solid component. The left ovary was normal in size (Figure 2).

**Figure 3:**
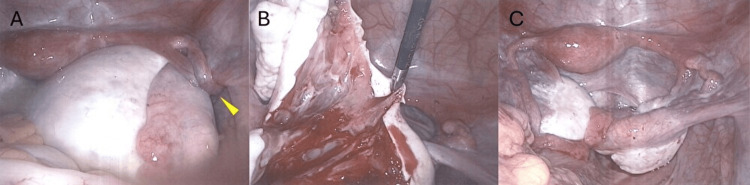
Intraoperative findings. (A) Markedly enlarged right ovary, exceeding the size of a goose’s egg, with a 360-degree torsion of the ovarian pedicle. The arrowhead indicates the site of torsion. (B) After cortical incision of the right ovary. No tumorous lesion is observed; instead, the ovarian parenchyma appears markedly edematous, with serous fluid leaking from the stroma. (C) End of surgery. Following detorsion and partial resection of the right ovary, the ovarian size has decreased substantially and has nearly returned to normal.

Serum tumor markers were within normal limits: CA125 was 12 U/mL (normal limits, ≤35 U/ml), CA19-9 was 8 U/mL (normal limits, ≤37 U/ml), and carcinoembryonic antigen was <1.7 ng/mL (normal limits, <5 ng/ml). The imaging findings were highly suggestive of MOE. Ultrasonography demonstrated a solid-appearing enlarged ovary with peripheral displacement of follicles and mottled hypoechoic areas consistent with stromal edema, while MRI showed a markedly enlarged ovary with edematous stroma exhibiting high signal intensity on T2-weighted images and follicles compressed toward the periphery by accumulated stromal fluid. Based on these characteristic radiologic features, MOE secondary to ovarian torsion was strongly suspected rather than an ovarian neoplasm. However, because the patient continued to experience abdominal pain and a definitive diagnosis could not be established preoperatively, surgical intervention was planned. Laparoscopic surgery revealed the right ovary enlarged to beyond the size of a goose’s egg, with a 360-degree torsion of the ovarian pedicle (Figure 3A). The left ovary appeared normal. After detorsion, a cortical incision was made to evaluate the internal structure. No tumorous lesion was identified; instead, the ovarian parenchyma appeared markedly edematous (Figure 3B). As the incision deepened, transparent serous fluid leaked from the ovarian stroma. For diagnostic confirmation, a small portion of the right ovary was resected by wedge resection, and hemostasis was secured. Notably, following detorsion and wedge resection, the swollen right ovary had already begun to decrease in size and had nearly returned to normal dimensions by the end of the procedure (Figure 3C).

**Figure 4:**
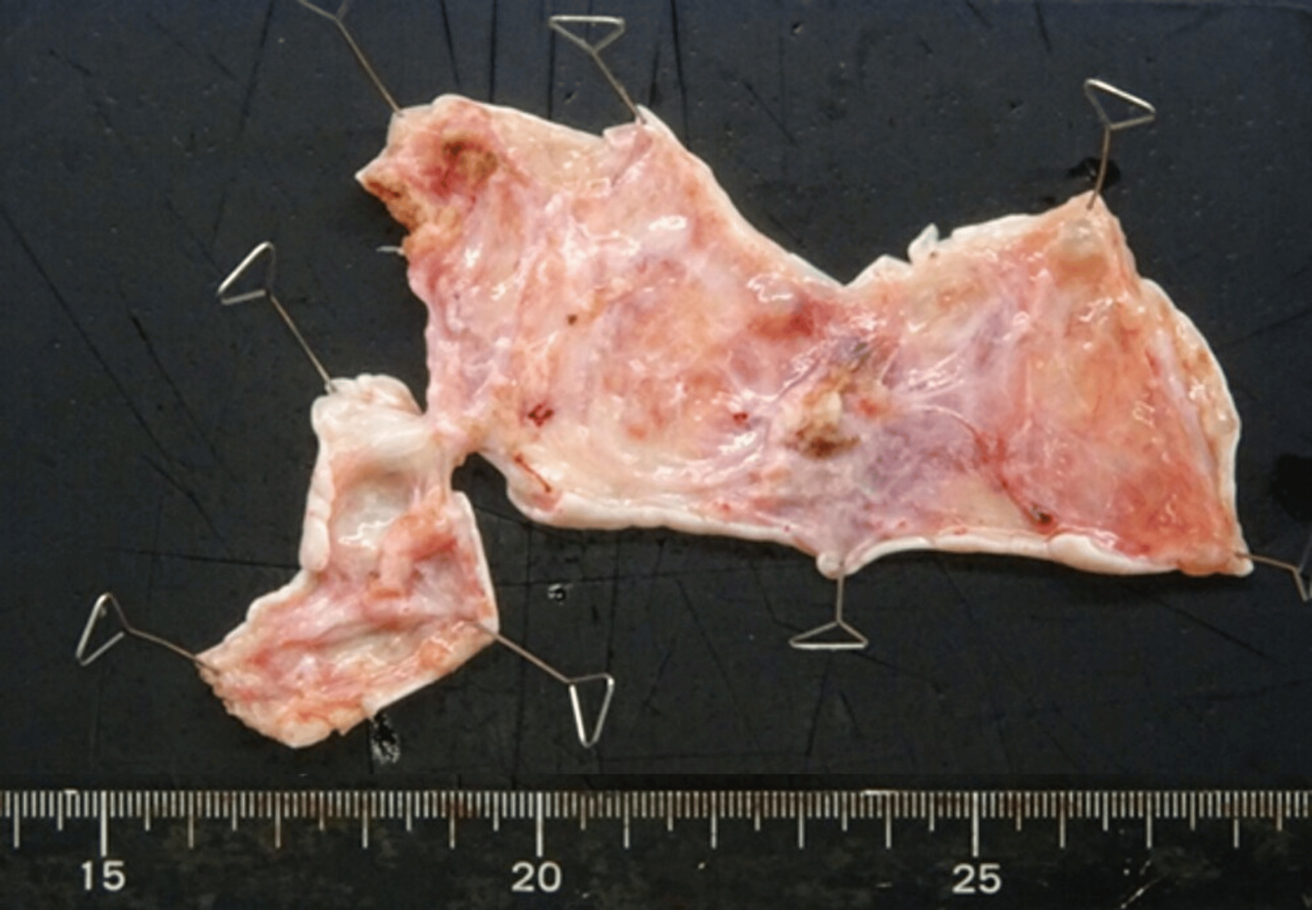
Gross findings of the resected ovarian specimen. The specimen exhibits diffuse stromal edema without a defined mass, and the cut surface is watery, consistent with massive ovarian edema.

The postoperative course was uneventful, and the patient was discharged on postoperative day four. The macroscopic examination of the resected ovarian tissue revealed a diffusely swollen ovary with edematous stroma and no identifiable tumorous lesion (Figure 4).

**Figure 5:**
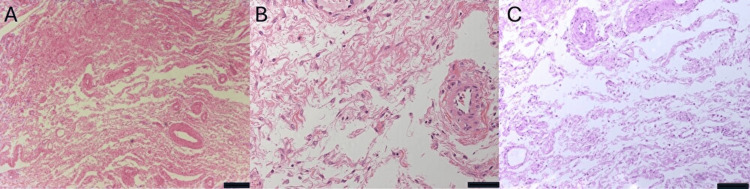
Histopathological findings of the resected portion of the right ovary (hematoxylin and eosin staining). Normal follicular architecture is preserved, and marked stromal edema is present. (A) Scale bar, 200 µm. (B) Scale bar, 100 µm. (C) Scale bar, 50 µm.

Histopathological examination demonstrated preservation of normal follicular architecture with marked stromal edema, consistent with MOE (Figure 5).

**Figure 6:**
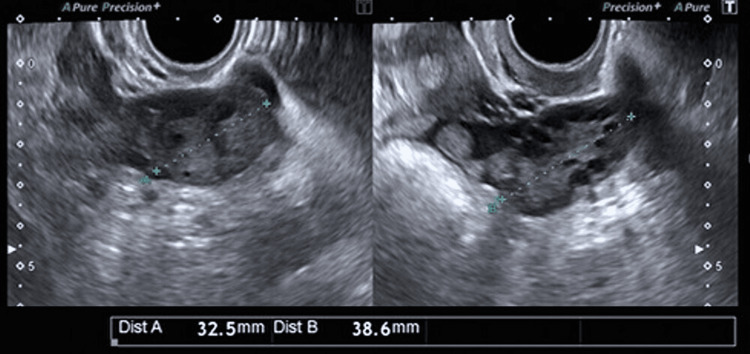
Follow-up transvaginal ultrasonography one month after surgery. The right ovary (left image) has decreased in size to 32.5 mm and returned to normal size.

At the one-month postoperative follow-up, transvaginal ultrasonography confirmed that the right ovary had returned to normal size (Figure 6).

## Discussion

MOE is an uncommon benign condition characterized by marked enlargement of the ovary due to the accumulation of stromal fluid while preserving normal follicular architecture. Since its first description in 1969 [1], MOE has remained a diagnostic challenge because its clinical and radiologic manifestations often mimic those of solid ovarian neoplasms. Most affected patients are adolescents or young women, highlighting the importance of accurate diagnosis to avoid unnecessary oophorectomy and preserve fertility [6].

The predominant mechanism proposed for MOE is partial or intermittent torsion of the ovarian pedicle, which impairs venous and lymphatic drainage while maintaining arterial inflow, ultimately leading to progressive stromal edema without ischemic necrosis [1,7]. In the present case, a 360-degree torsion of the right ovarian pedicle was identified intraoperatively, supporting torsion as the precipitating factor.

Radiologic evaluation plays a crucial role in preoperative diagnosis. Ultrasonography typically demonstrates a solid-appearing enlarged ovary with multiple peripheral follicles [8], whereas MRI shows high T2 signal intensity within the edematous stroma and peripheral compression of follicles [4,9]. These findings have been recognized as characteristic but are not exclusive to MOE, as they may overlap with those of ovarian fibroma, polycystic ovary, cystadenoma, and metastatic carcinoma [4,10]. In our patient, both ultrasonography and MRI revealed classic findings of MOE, enabling preoperative suspicion of the diagnosis. Furthermore, preoperative suspicion of MOE was strengthened by the fact that our team had previously encountered and published a case of massive ovarian edema [5], which enhanced our familiarity with its characteristic imaging and intraoperative features.

Surgical findings in MOE classically reveal a markedly enlarged ovary with edematous stroma and the cut surfaces appear grey-white, watery, and gelatinous [1,7,11]. In our case, the right ovary not only exhibited pronounced edema but also demonstrated dramatic reduction in size after detorsion and wedge resection, a finding that further supports MOE as a reversible condition when promptly treated. This intraoperative shrinkage is a hallmark feature, as demonstrated in our previously reported case [5], and may assist clinicians in confirming the diagnosis during surgery.

The management of MOE should prioritize ovarian preservation, particularly in young women. Historically, misdiagnosis has led to unnecessary oophorectomy; however, growing awareness and improved imaging have facilitated more conservative approaches. Importantly, a large review reported that among 177 published cases of MOE, 145 patients (81.9%) underwent adnexectomy and 12 patients (6.8%) underwent hysterectomy with bilateral salpingo-oophorectomy, whereas only 20 patients (11.3%) received conservative treatment [6]. This striking imbalance highlights the strong tendency toward overtreatment and emphasizes the need for heightened awareness of MOE as a benign, fertility-preserving condition. Wedge resection has been reported as a preferred treatment option for MOE [7]. In our patient, conservative laparoscopic surgery with detorsion and diagnostic wedge resection was successful, and postoperative recovery was uneventful. Follow-up ultrasound confirmed complete restoration of ovarian morphology within one month, reinforcing the suitability of fertility-sparing management.

## Conclusions

MOE is a rare but important clinical entity that can closely mimic ovarian neoplasm. Early recognition and fertility-preserving surgical management are crucial. The present case demonstrates the characteristic radiologic and intraoperative features of MOE and underscores the importance of considering this diagnosis in young women with ovarian enlargement and suspected torsion.
